# FollowTheSutures: Piloting a new way to administer onabotulinumtoxinA for chronic migraine

**DOI:** 10.1177/03331024211067775

**Published:** 2022-02-15

**Authors:** Lars Jacob Stovner, Knut Hagen, Erling Tronvik, Gøril Bruvik Gravdahl, Rami Burstein, David W Dodick

**Affiliations:** 1 Department of Neuromedicine and Movement Science, NTNU Norwegian University of Science and Technology, Trondheim, Norway; 2 Norwegian Advisory Unit on Headache, Department of Neurology and Clinical Neurophysiology, St Olavs University Hospital, Trondheim, Norway; 3 Clinical Research Unit Central Norway, St. Olavs Hospital, Trondheim, Norway; 4 Department of Neurology, Mayo Clinic, Scottsdale Arizona; 5 Department of Anesthesia, Critical Care and Pain Medicine, Beth Israel Deaconess Medical Center, Boston, MA, USA; 6 Harvard Medical School, Boston, MA, USA

**Keywords:** Cranial sutures, pilot study, injections

## Abstract

**Background:**

Anatomical and experimental data indicate that onabotulinimtoxin A could be more efficient and cost-effective for treating chronic migraine with injections targeting the cranial sutures, where collaterals from the meninges penetrate the skull.

**Methods:**

A new injection paradigm (FollowTheSutures) was tested for safety, tolerability and feasibility in a Phase II, open-label, non-controlled, single-center pilot study. Ninety units of onabotulinimtoxin A (Botox®), were injected in 18 sites over the area of the cranial sutures. Adverse events and potential beneficial effects were recorded in a headache diary at least 4 weeks before, and for 12 weeks after the injections. A higher dilution than normal of onabotulinimtoxin A was used to get better diffusion.

**Results:**

Nineteen (of 20 included) women with chronic migraine received the injections and were evaluable. There was only one treatment-related adverse event (reduced power of chewing for some weeks). Otherwise, the procedure was overall well tolerated. Patients improved on most efficacy parameters after the injections. There was little or no effect on glabellar or forehead lines.

**Conclusions:**

The protocol was safe and well tolerated. Lower risk of unblinding due to the absence of cosmetic effects should make the injection procedure well suited for a large, randomized, placebo-controlled study. If efficacy is confirmed, it will be markedly less costly than the standard procedure.

Trial registration: EUDRACT (2017-002516-13), ClinicalTrials.gov (NCT03543254).

## Introduction

During the last 10 years, injection of onabotulinumtoxinA (BoNT-A) in pericranial and neck muscles has become an established and regulatory-approved method of treating chronic migraine (CM) around the world. The effect in CM was shown in a pooled analysis of two large studies (PREEMPT 1 and 2) (1–3), including almost 1400 patients who had injections of five units of BoNT-A or placebo in 31 sites in the head and neck (PREEMPT injection paradigm), and in addition up to 40 units at different sites of maximal pain. The fact that many patients may experience cosmetic effects with loss of forehead and glabellar lines with the PREEMPT injection paradigm raises the question of unblinding. In addition, the most common adverse events were related to the muscles injected in the forehead and neck, including neck pain, muscular weakness, and eyelid ptosis. In general, the safety and tolerability of the procedure was good.

A major disadvantage with BoNT-A treatment for CM is the cost, which for each treated patient exceeds US$6300 annually (https://www.biospace.com/employer/506492/prime-therapeutics/). In addition, each quarterly injection cycle involves 31–37 intramuscular injections in seven head and neck muscles. This can be painful, especially in a patient population where cutaneous allodynia occurs in approximately 93% of patients (4).

The pathophysiological mechanism underlying the efficacy of BoNT-A in CM is not clearly understood, but blockade of SNAP-25 mediated exocytosis of proinflammatory neuropeptides including CGRP and Substance P and excitatory neurotransmitters including glutamate may be one plausible mechanism (5). BoNT-A also decreases the insertion of pain-sensitive ion channels such as transient receptor potential cation channel subfamily V member 1 (TRPV1) into the membranes of nociceptive neurons.

Still, it has been a puzzle how the administration of the toxin in the pericranial and neck muscles outside the skull affects migraine headache, which is believed to involve activation of meningeal nociceptors inside the calvarium. An answer to this may be the demonstration of intracranial sensory fibers in the meninges that send collaterals, which pass through the skull bones into extracranial tissue through the sutures and emissary vein channels, to innervate the periosteum and extracranial muscles in both mice, rats and humans (6–9).

It has also been shown that BoNT-A can inhibit mechanical nociception in peripheral trigeminal neurons in rodents (10), and that injection of BoNT-A extracranially in the region of the sagittal and lambdoid sutures can suppress nociceptor response to stimulation in the meninges (dura mater) (11). The effect of injection near the sutures was more pronounced than when it was injected in the temporalis and neck muscles.

These preclinical data suggest that a “follow-the-suture” (FTS) approach to injections of BoNT-A in patients with CM could represent an effective and less invasive and costly injection strategy than the currently employed PREEMPT injection paradigm. The aim of the present pilot study was to develop an FTS injection paradigm and evaluate its feasibility, tolerability, and acceptability among patients with CM in an open-label pilot study.

## Method

The study was performed as an open-label, non-controlled, single-arm and single-center phase II study, at the outpatient clinic of the Department of Neurology and Clinical neurophysiology at St. Olavs Hospital, Trondheim, Norway. Another part of the study was also planned at the Mayo Clinic, Scottsdale, AZ, USA, but because the study there had not been started before the COVID-19 pandemic, and therefore would have had to be postponed for an extended period of time, the study was performed only in Trondheim. The trial was registered as EUDRACT (2017-002516-13), ClinicalTrials.gov (NCT03543254) and approved by the Ethics Committee of Central Norway (REC Central). The study was funded by the hospital and university departments in Trondheim.

**Figure 1.**
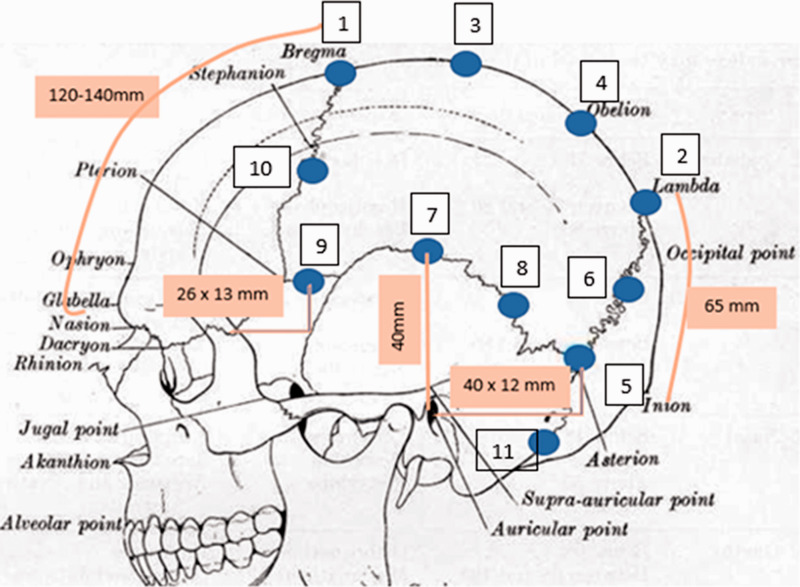
Locations of OnabotulinumtoxinA injections. For some points (1,2,5,7,9) the distance from easily identifiable landmarks is given. Points 6,8,10 were midway between two other points (5 and 2, 5 and 7, and 1 and 9), and 3 and 4 were distributed evenly between 1 and 2. Five units of BoNTA were given at each point.

Potential study participants were identified among the regular outpatients at the Department of Neurology at St. Olavs hospital. Potential participants had been sent an email with the informed consent form, which was signed at the screening visit where a detailed medical history and medical examination was performed and eligibility was determined. Patients were then given instructions in completion of a paper headache diary, where they recorded pain intensity and duration, concomitant migraine symptoms (nausea, vomiting, phono- and photophobia), acute medication (type, number of doses) and work absence (yes/no/not relevant). Also, they were asked to record any adverse events. After 7–14 days, they received a telephone call from a nurse asking about adverse events and concomitant medication and reminding them to maintain the daily diary. After a baseline period of at least 28 days, diaries were reviewed at a treatment visit, and each participant received injections according to the described procedure (Figure 1). Women of child-bearing potential were required to have a negative pregnancy test before the injections. The duration of the injection procedure was measured with a stopwatch and immediately after the injections, the patient was asked to record on a VAS scale (0–10) the level of pain of the procedure and the investigator recorded the degree of bleeding (no, mild, moderate, marked) and any other injection-related adverse events (AEs).

Patients were asked to keep a headache diary for another 12 weeks or more, before the end of the study visit. During this period, they received a call from the study nurse at weeks 2 and 9, when they were reminded to keep the headache diary, and were asked about AEs and concomitant medication. They were also asked whether they had noticed any change in their ability to make forehead wrinkles (no, little, moderate, marked, full paralysis).

### Inclusion and exclusion criteria

Inclusion criteria were: a) Men or women between 18 and 64 years of age with CM, as defined in the ICHD-3 beta version (12); b) CM should have been present for at least 6 months prior to evaluation for study inclusion; c) for women of child-bearing potential there must be no pregnancy or planned pregnancy during the study period, and use of highly effective contraception; d) patients must have signed an informed consent form. 

Exclusion criteria were: a) diseases that are contraindications for use of BoNT-A (myasthenia gravis, Eaton-Lambert syndrome, amyotrophic lateral sclerosis, other diseases interfering with neuromuscular function) or allergy to BoNT-A; b) another primary or secondary headache disorder, including medication overuse headache (MOH). This means that at least one attempt to withdraw acute medication should have been performed earlier, but without success. c) Severe depression or other psychiatric disorder that may interfere with the treatment; d) abuse of alcohol or illicit drugs; e) use of more than one headache preventive medication or change in type and dose of preventive medication ≤28 days before start of the baseline period. f) Previous exposure at any time to any botulinum toxin serotype; g) infection at any injection site(s); h) the patient having received extracranial nerve block, cervical facet injection, or another interventional procedure for headache within the previous 3 months; i) use of opioids or barbiturate containing medication(s) ≥10 days per month within the preceding 3 months; j) participation in another trial that might affect the current study; k) if in the opinion of the investigator, the patient should not participate (e.g. if they were not able to comply with study procedures).

### Endpoints

The primary endpoint was number of AEs recorded during the study.

Some secondary endpoints were also related to the injection procedure including bleeding and pain associated with the injections.

Secondary endpoints also included measures of potential efficacy: Compared to baseline, change in weeks 5–8 of: a) moderate/severe headache days (main efficacy variable), defined as headache lasting ≥4 h with at least moderate intense pain; b) headache days, defined as a day with headache lasting ≥4 h with mild, moderate, or severe pain; c) migraine (definite or probable) headache days; d) acute headache pain medication intake (all categories); e) triptan intake. The period between week 5 and 8 was chosen because the effect of BoNT-A starts after 1–2 weeks and tapers off after 10–12 weeks (see, e.g. (13)). In addition, we included an endpoint f) moderate/severe headache days during the 12 weeks after injection.

### Preparation of BoNT-A

One hundred units of BoNT-A (Botox®, Allergan Inc) were dissolved in 4 ml (cc) of isotone saline water (9 mg/ml), distributed in four syringes. This is half the usual concentration, allowing for better diffusion in order to reach the target structures (14).

Five units (0.2 ml) were injected subcutaneously, and down to but not penetrating the galea aponeurotica, at each site (Figure 1). Needle size: 30 gauge, 1.3 cm length.

### Injection sites

In the study of rat skulls, innervation has been found to be particularly dense in the region of the occipitomastoid suture around the attachment of the splenius and longissimus capitis muscles, and in the human skull in the region of the squamous suture, between the parietal bone and the squamous part of the temporal bone (6,7,9). However, in these studies, not all sutures were investigated, and in a study of adult mice, a rich innervation by fibers passing from inside to outside the skull has been demonstrated in all sutures (6). Based on these observations, 18 injections of five units of BoNT-A (total 90 units) were chosen (Figure 1). 

### Statistical analysis

For the efficacy variables, mean values with standard deviations for each 4-week period, change (in %) between baseline and each 4-week period, and the whole 12-week period after injection were calculated. In patients who had recorded more than 4 weeks in the baseline period, only the first 28 days were considered. Statistically significant change from baseline was tested with a paired t-test. *p* ≤ 0.05 was considered significant. Patients with ≥50 reduction in one of the periods compared to baseline were considered responders. In the protocol, the primary efficacy endpoint was defined as the change in week 5–8.

## Results

Twenty patients with chronic migraine were included, all women, between May 2018 and October 2019. The mean age was 40 years (SD 9, range 19–58), and 19 received BoNT-A injections, the last patient being lost to follow up after the baseline period. Nineteen patients completed the study and provided data. All used some acute medications, and 18 of them used triptans. Five of them used one preventive medication for migraine (candesartan, topiramate, amitriptyline, mirtazapine, lamotrigine) during the study. Seventeen had tried at least one type of preventive medicine in the past; 14 had tried at least two, and 10 had tried three or more. Five were smokers, and one was a previous smoker.

### Injection

In 16 patients, the time used for injecting the medicine, measured with a stopwatch (not including the preparation of the toxin and syringes) was on average 5 min 47 sec (SD 1 min 2 sec, range 3 min 46 sec to 7 min 22 sec). In three patients, it was not measured.

All 19 patients noted some pain during the injections, the average being 2.1 (SD 0.8, range 1–4) on a 0–10 VAS scale. In eight patients, the investigator noted some bleeding at the site of injection, seven of them mild and one moderate.

### Adverse events

All AEs occurring during the study are listed in Table 1. Only one patient had an AE that most likely was caused by BoNT-A (reduced power of chewing), but with no functional impairment. It occurred during the first month after injection, but the exact duration was not given. Otherwise, patients reported common infections and various bodily pains, including neck pain, both during baseline and in the treatment period, but none were considered related to the injections or the toxin.

**Table 1.** Adverse events during baseline and the first 3 months after injection.

Adverse event	Baseline	Week 1–4	Week 5–8	Week 9–12
Decreased chewing power		1		
Common cold	6		1	1
Influenza		1		
Sinusitis	3		1	1
Neck pain	2	1		1
Low back pain		1		
Stomach pain	1	1	1	2
Other bodily pain		1		1
Diarrhoea				1
Exanthema	1	1	1	1

### Effect on forehead wrinkles

Seven days after injection, two patients noted mild difference in their ability to wrinkle their forehead. No patients noticed this difference after 6 weeks.

### Efficacy measures

Efficacy measures are shown in Table 2. Days with moderate to severe headache were significantly reduced compared to baseline in week 5–8 after injection (9%, *p* ≤ 0.05), and so were days with headache lasting ≥4 h (26%, *p* ≤ 0.01). All parameters, including acute medicine and triptan doses, and days with work absence, were reduced in the first two periods after injection and also in period 3 with the exception of days with moderate to severe headache. There were large variations in all parameters (cf. standard deviations) but 32% experienced ≥50% reduction in moderate to severe headaches in the second and third periods. Regarding migraine day reduction, the responder rates during the second and third periods were 37% and 47% respectively, while 42% and 32% experienced a significant reduction in acute medication use and 39% and 67% experienced a significant reduction in triptan use. Days absent from work was particularly reduced in the first and second period after injection (mean 66% and 44%).

**Table 2.** Compared to baseline, change (%) in the three 4-week periods after injection and number and percentage of 50% responders.

Endpoint	Baseline (mean (SD) n of days/doses)	Mean change from baseline in % (SD) in week				50% responders in week (n, %)		
		1–4	5–8	9–12	1–12^1^	1–4	5–8	9–12
Days with moderate to severe headache	12.9 (6.1)	−4.6 (59.6)	−9.2 (63.8)*	10.6 (147.8)	−1.1 (80.1)*	3 (16)	6 (32)	6 (32)
Days with headache lasting ≥4 h	18.1 (5.7)	−15.1 (34.0)*	−23.7 (28.4)**	−24.0 (29.2)**	−21.0 (25.6)**	2 (11)	3 (16)	5 (26)
Days with migraine	10.4 (6.5)	−7.5 (72.0)*	−3.4 (85.6)	−9.3 (98.2)*	−6.7 (77.8)	5 (26)	7 (37)	9 (47)
Days with definite or probable migraine	17.7 (5.0)	−21.8 (31.6)**	−26.9 (33.2)**	−25.6 (31.7)**	−24.8 (26.7)	4 (21)	5 (26)	4 (21)
Doses of acute medicines	24 (18)	−8 (50)	−28 (46)**	−18 (36)	−22 (37)*	5 (26)	8 (42)	6 (32)
Doses of triptans^2^	10 (6)	−10 (112)*	−27 (50)*	−35 (46)*	−25 (50)*	7 (39)	7(39)	12 (67)
Absence from work (n of days)	1.6 (2.0)	−66 (161)*	−44 (134)	−3 (119)	−38 (30)			

^1^Mean/4 weeks.

^2^Computed among the 18 patients using triptans.

*p ≤ 0.05, **p ≤ 0.01, compared to baseline (paired t-test).

SD: Standard deviation.

## Discussion

In this open label pilot study utilizing a novel follow-the-suture injection paradigm with 90 units and 18 injection sites, there was a significant reduction in moderate and severe headache days, days with headache lasting ≥4 h, days with ICHD-3 beta migraine and probable migraine, acute medication use, triptan use, and absenteeism from work during weeks 5–8. Overall, the injection paradigm was well tolerated with the only treatment related adverse event occurring in one patient who noted partial reduction in chewing power without problems with eating. This was probably related to the injections in the temporalis muscles. Neck pain and paresis of neck muscles, which may be found with the PREEMPT procedure, is avoided. Weakness of frontalis and glabellar muscles with cosmetic changes and ptosis are also mostly avoided with this injection paradigm, although it is a limitation that ability to make forehead wrinkles was only based on patients’ self-assessment at home and reported on the phone. The procedure was easy to perform, taking on average less than 6 min. There was a clear learning curve, so with experience, when it is no longer necessary to measure distances from the anatomical landmarks, this time can likely be substantially reduced. None of the patients found the procedure very painful (maximum degree 4 on a 0–10 scale). Hence, the procedure seems to be safe, effective, easy to perform, well tolerated, less burdensome (18 injections versus 31–39 injections), and possibly more cost-effective (90 units vs. 155–195 units) compared to the PREEMPT technique. Only two patients thought there might be a little reduction in ability to make forehead wrinkles one week after the injection. Hence, in a potential future controlled and blinded trial, effective blinding may be achieved.

A definitive treatment effect is impossible to evaluate properly in an open-label study. There was significant improvement in five of seven efficacy endpoints during weeks 5–8 after the injection, which was chosen *a priori* as the most relevant period. Of course, this change may be due to a placebo effect or a period effect. The latter occurs because people with migraine may have naturally occurring fluctuations in their headache frequency, tend to seek help when they are in a more severe phase, and the headache is then likely to improve in the months afterward.

One may try to compare results with those of PREEMPT (3), the only large randomized controlled trials with BoNT-A for chronic migraine. In that study, patients were followed in a blinded phase for 24 weeks, and the primary endpoint was measured after two quarterly injections during weeks 21–24. In this period, the change in mean headache days was −42%, mean migraine days −43% (including both definite and probable migraine), and mean moderate to severe headache days were −43%. This is substantially higher than the comparable figures for the primary efficacy endpoints in the present study (−24%, −27% and −9%). Comparing the percentage of 50% responders with regard to headache days, this were 48% in PREEMPT and 26% in the present study. However, there are many differences between the two studies that make a comparison difficult. Compared to the present study, patients in PREEMPT had more days with migraine (19.9 vs. 10.4) in the baseline period, and the efficacy data in PREEMPT occurred after two injections, whereas in this study we only evaluated outcomes after one injection. In the PREEMPT trials, the efficacy increases over time and after the second injection, as is seen in most migraine preventive trials. On the other hand, the placebo effect may be larger when there is no placebo arm. This, and the fact that a marked improvement was seen in the placebo arm of the PREEMPT study, make speculations about an effect, or lack of effect, in the present study futile.

As to the injection sites chosen, these were easy to identify. However, when performing the procedure, it appeared that the points were not as evenly distributed as it appears in Figure 1. In particular, the distance along the coronal suture was longer than in other locations. In a potential future study, it might be preferable to add one point at each side here, possibly with the last two doses of the 100-unit vial.

One should also consider the possibility that a higher dose, possibly of the same magnitude as in the PREEMPT study (155–195 units) but given in accordance with the present paradigm or more densely along the sutures, might have given an even better effect than with the PREEMPT injection paradigm. A future study could investigate the effect of both a lower and a higher dose, and over several injection cycles, not least because it has been shown that wear-off of effect is lower with a higher dose (15) but also because the benefit may increase over subsequent injection cycles (3).

The main weakness of the present study, in addition to being open label, is the low number of participants, which makes it prone to incidental findings. Originally, it was planned for twice as many, in two centers, but this was prohibited by the pandemic. Also, one would have wished to see the effect of more injection cycles, as was done in the PREEMPT study.

## Conclusion

Administering BoNT-A over the cranial sutures is based on recent elegant anatomical and animal experimental studies. It is practical, well tolerated, and with less risk of unblinding in the context of placebo-controlled clinical trials. This small open-label study suggests the possibility of a treatment effect, but confirmation will certainly require an adequately sized, multi-center, randomized placebo-control study. A head-to-head trial comparing outcomes of FollowTheSuture (FTS) and PREEMPT injection paradigms would be necessary to evaluate the comparable efficacy, tolerability, and any differences in patient acceptance.

## Article highlights

- Based on preclinical studies, injecting onabotulinumtoxinA over the cranial sutures for chronic migraine could be more effective and cost saving compared to the standard procedure.
- This open-label, pilot study in 19 women indicate that a new injection paradigm (FollowTheSutures) may be tolerable, safe and effective.
- Fewer cosmetic effects on forehead lines suggest that the paradigm may be well suited for a larger blinded and placebo-controlled study.
